# Immunological mechanisms and precision stratification in male infertility: from testicular immune privilege and danger signal amplification to seminal immune biomarkers and mechanism-tailored intervention

**DOI:** 10.3389/fimmu.2026.1806683

**Published:** 2026-05-12

**Authors:** Jiedong Zhou, Shian Hu, Yong Ouyang, Dong Yang, Zhi Su, Min Liu

**Affiliations:** 1 The First Affiliated Hospital of Gannan Medical University, Ganzhou, Jiangxi, China; 2 Department of Urology, the First Affiliated Hospital of Gannan Medical University, Ganzhou, Jiangxi, China; 3 Department of Reproductive Medicine, the First Affiliated Hospital of Gannan Medical University, Ganzhou, Jiangxi, China

**Keywords:** male infertility, immune endophenotype, seminal immune biomarkers, testicular immune privilege, blood-testis barrier, sperm DNA fragmentation, inflammasome, precision medicine

## Abstract

Male infertility presents significant heterogeneity, yet traditional assessments primarily rely on semen parameters and endocrine, imaging, and genetic screening. Although these methods can identify some definitive etiologies, a mechanistic explanatory gap persists in large populations with mild to moderate oligoasthenoteratozoospermia, elevated sperm DNA fragmentation, or recurrent adverse reproductive outcomes. The testis maintains a dynamic immune-privileged balance by balancing infection defense against tolerance to post-pubertal germ cell antigens. In this context, infections, varicocele-induced hypoxia or thermal stress, metabolic abnormalities, and environmental factors, trigger innate immune recognition via PAMPs or DAMPs. Notably, most mechanistic insights discussed are derived from animal models and *in vitro* studies; direct high-level clinical evidence in humans remains limited, and the proposed framework requires prospective validation. This is proposed to drive myeloid amplification, complement cascades, and inflammasome activation, which may in turn mutually reinforce oxidative stress. Based on current evidence, this may consequently compromise blood-testis barrier integrity, cause immune tolerance thresholds to decline, and induce tissue remodeling, collectively impairing the spermatogenic microenvironment, sperm maturation, and DNA integrity. This article reviews these immunological mechanisms and proposes a hypothesis-driven intervention framework driven by immune endophenotypes, aiming to provide an actionable clinical roadmap for the precision management of male infertility.

## 1 Introduction: reconstructing the “etiological map” of male infertility with immunology

Male infertility is a critical issue in reproductive health that exhibits pronounced clinical heterogeneity (1). Although routine semen analysis, hormonal profiling, imaging, and genetic screening can identify some clear causes, a considerable proportion of patients in real-world practice remain classified as having “idiopathic infertility” because the current framework does not provide a clear, interpretable, or actionable mechanistic diagnosis (2). This suggests that traditional assessments are more adept at detecting structural abnormalities, overt endocrine disorders, or directly detectable infections. They are less effective at identifying chronic low-grade damage, microenvironmental imbalance, or functional impairments (3).

Notably, male infertility does not in many cases correlate directly with significant abnormalities in routine semen parameters. For instance, some patients exhibit only mild or near-normal routine parameters but have elevated sperm DNA fragmentation (SDF), impaired mitochondrial function, reduced fertilization rates, poor embryo quality, or recurrent pregnancy loss (4, 5). Conversely, others show marked fluctuations in semen parameters that do not strictly correlate with reproductive outcomes. This inconsistent phenotype-outcome mapping indicates that relying solely on semen parameters as the core phenotype is insufficient for guiding individualized management (6). Instead, a mechanistic readout that more directly reflects the underlying pathological process is necessary. Such a readout would help explain why patients with similar semen profiles respond differently to the same intervention and why certain treatments may improve surrogate markers without improving live birth rates-a critical gap that is often overlooked in the literature.

Immunology may offer an integrative perspective to reframe this issue. The male reproductive system is a tightly regulated immune homeostatic system. The testis must maintain a dynamic equilibrium between anti−infection defense and tolerance to post−pubertal germ cell antigens (7). The epididymis and accessory glands are at mucosal interfaces and are constantly exposed to microbial and environmental pressures. Seminal components-including immune cells, cytokines, complement, and exosomes-both reflect local immune status and may actively regulate sperm maturation and function (8). In different etiological contexts, infections trigger innate immune recognition via pathogen-associated molecular patterns(PAMPs) (9), whereas hypoxia, thermal stress(e.g., from varicocele), metabolic disorders, and toxicant exposures tend to act through damage-associated molecular patterns(DAMPs) and reactive oxygen species(ROS) signaling (10). Importantly, both pathways have been suggested to converge into sustained inflammation maintained by myeloid amplification, complement cascades, and inflammasome activity, processes that may mutually reinforce oxidative stress. This loop ultimately compromises blood-testis barrier(BTB) stability and lowers immune tolerance thresholds, manifesting as impaired sperm function and DNA integrity.

## 2 Clinical pathways and the “mechanistic gap”: practical problems for an immunological framework to address

### 2.1 Strengths and blind spots of current diagnostic pathways

Standard evaluation includes systematic history and exposure assessment, physical examination (focusing on testicular volume and varicocele), semen analysis (at least two samples), hormonal profiling, infection screening, and, in appropriate populations, genetic testing and scrotal ultrasound (11, 12). This pathway has a high detection rate for structural or endocrine causes and provides a basis for treating overt infections.

Blind spots primarily emerge in populations dominated by “chronic low-grade inflammation and oxidative damage.” These individuals may lack clear evidence of infection, show no specific imaging abnormalities, and have near-normal hormonal profiles. Nevertheless, persistent sperm dysfunction exists (13). In such cases, empirical antioxidant or anti-inflammatory therapy alone often involves prolonged courses with difficulty determining whether the underlying pathological process is truly altered.

### 2.2 Why immune indicators can serve as a “process readout”

Semen parameters can fluctuate significantly and are influenced by the observation window. In contrast, immune-inflammatory modules are more closely linked to the pathological process. Examples include inflammatory cell load, cytokine/chemokine profiles, myeloid amplification-related proteins(degranulation products, complement fragments), and oxidative damage readouts(ROS, DNA oxidation damage) (14, 15). These indicators are more sensitive in reflecting whether “local immune homeostasis is disrupted.”

When standardized and reproducibly measurable, these indicators hold potential for two clinical tasks: stratifying patients based on immune endophenotypes and serving as early monitoring tools post-intervention to assess whether the treatment direction is correct.

### 2.3 Evidence organization strategy: mechanistic narrative and clinical evidence loop

To avoid the critique of “rich mechanistic elaboration but insufficient supporting evidence,” this article incorporates representative clinical research evidence at key junctures. Focus is placed on two areas most amenable to forming evidence loops: sperm functional endpoints(exemplified by SDF) and longitudinally monitorable seminal immune biomarkers. The intervention strategy section will prioritize evidence from randomized controlled trials or systematic reviews/meta-analyses, clearly stating their outcome hierarchy(mechanistic indicators, sperm function, pregnancy/live birth) (4, 16).

This article proposes a unifying immune-inflammatory loop initiated by PAMPs/DAMPs, sustained via myeloid amplification-complement/inflammasome activity, and mutually reinforced with oxidative stress. This explains the common pathway leading to impaired sperm functional endpoints across different etiologies. It also proposes four immune endophenotypes based on clinically accessible readouts (seminal immune biomarkers), organizing heterogeneity via “entry signal +amplification mode+primary damage.” Finally, it provides a mechanism-matched intervention and hierarchical efficacy evaluation loop (mechanism, function, outcome) based on endophenotypes, emphasizing SOPs and repeated measurements to enhance reproducibility and verifiability (**Box 1**).

Box 1 **Three translational contributions of this article.**

This article proposes a unifying immune-inflammatory loop initiated by PAMPs/DAMPs, sustained via myeloid amplification‑complement/inflammasome activity, and mutually reinforced with oxidative stress. This explains the common pathway leading to impaired sperm functional endpoints across different etiologies. It also proposes four immune endophenotypes based on clinically accessible readouts (seminal immune biomarkers), organizing heterogeneity via “entry signal + amplification mode + primary damage.” Finally, it provides a mechanism-matched intervention and hierarchical efficacy evaluation loop (mechanism, function, outcome) based on endophenotypes, emphasizing SOPs and repeated measurements to enhance reproducibility and verifiability.

## 3 Testicular immune privilege: from isolation to controlled homeostasis

The BTB, formed by tight junctions between Sertoli cells, relatively isolates post-meiotic germ cells from systemic immunity and is widely considered a crucial anatomical basis for immune privilege. Its core significance lies in the fact that the multitude of new germ cell antigens appearing post-puberty may not have undergone central tolerance selection. Without seclusion and local tolerance mechanisms, they could theoretically more readily induce immune attack (17). Importantly, the BTB does not completely block immunity but maintains antigen exposure at a low-risk level while permitting necessary immune surveillance (18). Beyond structural and nutritional support, Sertoli cells also participate in immune regulation by secreting regulatory mediators that influence local inflammatory thresholds and immune cell states (18). Resident macrophages contribute to homeostasis repair and limit excessive inflammation (19), and regulatory lymphocytes help maintain tolerance balance (20). This ecosystem generally maintains a low-inflammatory, repairable environment conducive to continuous spermatogenesis. Animal studies suggest that when BTB function is impaired or local tolerance thresholds decrease, opportunities for antigen exposure and immune infiltration increase, making sustained local inflammation more likely (21). Persistent inflammation exacerbates tissue damage via ROS and protease load, which in turn releases more DAMPs, further amplifying the immune response (7, 22). The ultimate manifestations include instability of the spermatogenic microenvironment, declining Sertoli cell function, disturbances in sperm maturation, and impaired DNA integrity (23).

## 4 Danger signals and innate immune amplification: the engine of a common terminal pathway across etiologies

### 4.1 PAMPs and DAMPs as convergent entry points

Infectious inflammation primarily uses PAMPs as triggers. This rapidly initiates innate immune recognition and drives strong inflammatory responses (24, 25). In contrast, non-infectious injuries more commonly activate immune recognition via DAMPs and ROS. Examples include varicocele-induced hypoxia and thermal stress, lipotoxicity from metabolic disorders, or mitochondrial damage caused by tobacco, heavy metals, or endocrine disruptors (26, 27).

A key unresolved question is whether DAMP-driven inflammation is inherently less severe than PAMP-driven inflammation or simply follows a slower, more chronic trajectory. Some studies suggest that chronic DAMP signaling is associated with a low-grade but long-lasting inflammatory state that is more difficult to resolve spontaneously (26), whereas others argue that the intensity of inflammation depends more on the cellular context and the specific DAMP involved than on the trigger type per se. This distinction has important clinical implications: therapeutic strategies targeting PAMP receptors (e.g., TLR4 antagonists) may not be effective in DAMP-dominant conditions, and anti-inflammatory approaches that work well for acute infections could be ineffective or even counterproductive in chronic sterile inflammation. It remains unclear whether these pathways operate similarly in humans; most evidence comes from rodent models or ex vivo human samples.

### 4.2 Myeloid amplification: from defensive response to sustained damage

Neutrophils and monocyte-macrophages are core executors of innate immune amplification. In the acute phase, they help clear pathogens and necrotic debris. However, under chronic stimulation-such as persistent DAMP release from varicocele or metabolic syndrome-sustained release of degranulation products, proteases, and ROS can contribute to bystander damage to Sertoli cells and germ cells (28, 29). Notably, there is ongoing debate regarding whether this myeloid activity is purely detrimental or partially adaptive. Some researchers argue that low-level myeloid activation may be necessary for tissue homeostasis and repair (30). while others contend that any sustained activation beyond a certain threshold may inevitably drive fibrosis and irreversible damage. Clinically, chronic myeloid activation often coincides with decreased sperm motility and increased DNA fragmentation, but direct causal evidence in humans remains limited.

### 4.3 Complement and inflammasomes: amplifiers and sustainers of the inflammatory loop

The complement cascade enhances chemotaxis, amplifies inflammation, and strengthens phagocytic clearance, serving as an important amplification mechanism that bridges infectious and sterile inflammation (31). Inflammasomes-particularly NLRP3-convert danger signals such as mitochondrial damage and ROS into stable pro-inflammatory output (e.g., IL-1βand IL-18), making chronic inflammation more difficult to resolve spontaneously (32).

A controversial area in the field concerns the relative contribution of complement versus inflammasome activation in male infertility. Some studies emphasize the dominant role of the NLRP3 inflammasome in varicocele-mediated testicular damage (9), whereas others find that complement activation products (e.g., C3a, C5a) correlate more strongly with SDF than inflammasome markers. It is likely that both systems operate in parallel, mutually reinforcing each other: increased inflammation can contribute to more tissue damage and DAMP release, which in turn further activates complement and inflammasomes, thereby maintaining a self-perpetuating inflammatory loop (33, 34). However, most of these data come from animal models or cross-sectional human studies; longitudinal interventional studies that track complement and inflammasome markers before and after treatment are sorely lacking.

**Figure 1:**
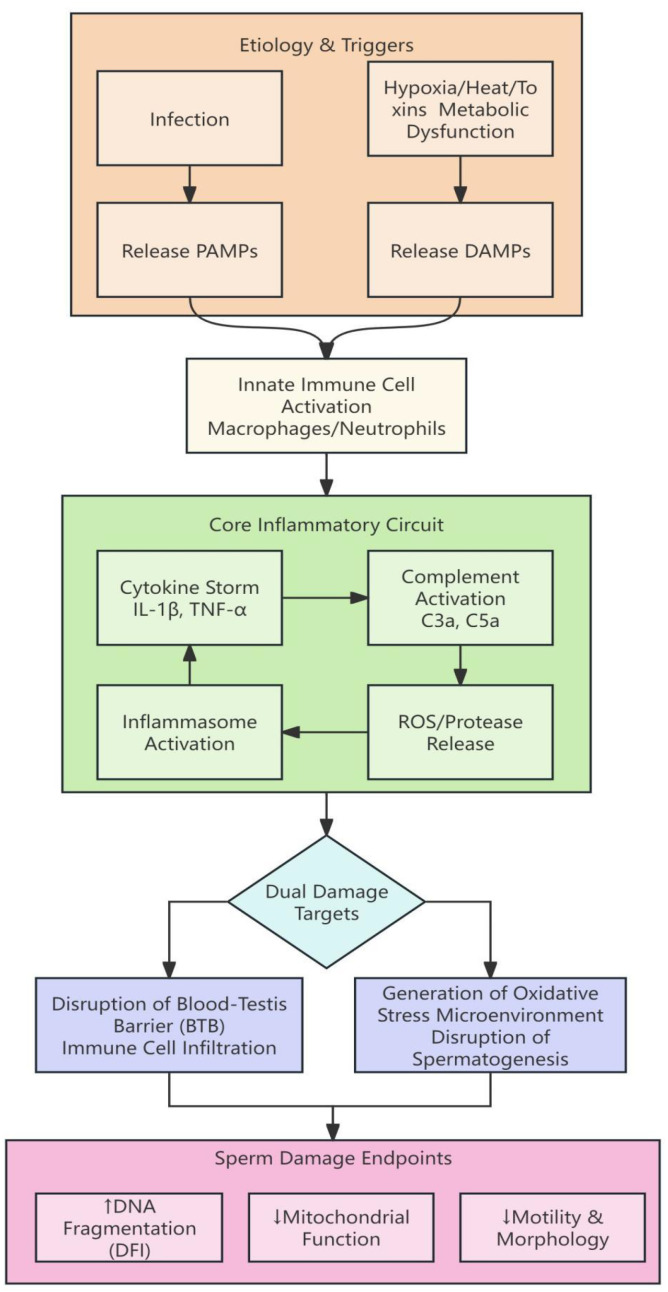
Common immuno-inflammatory pathway mechanism in male infertility. Multiple etiologies (infection, hypoxia/thermal/toxicant exposure, metabolic abnormalities) release PAMPs or DAMPs, activating innate immune cells (macrophages and neutrophils) within the male reproductive tract. This triggers a self-amplifying core inflammatory loop involving cytokine release, complement activation, ROS/protease production, and inflammasome activation. Sustained inflammation can contribute to dual damage: blood-testis barrier disruption with immune cell infiltration, and an oxidative stress microenvironment interfering with spermatogenesis. These insults ultimately converge on sperm damage endpoints, including increased DNA fragmentation, mitochondrial dysfunction, and impaired motility/morphology. PAMP, pathogen-associated molecular pattern; DAMP, damage-associated molecular pattern; ROS, reactive oxygen species; BTB, blood-testis barrier.

For instance, studies focusing on varicocele patients have consistently reported NLRP3 inflammasome activation as a dominant driver of SDF (35), whereas investigations in men with metabolic syndrome or obesity have found complement markers to be more strongly associated with sperm dysfunction (36). These contrasting findings may reflect etiology-specific immune pathways: varicocele primarily induces hypoxia and mechanical stress, which are potent NLRP3 triggers, while systemic low-grade inflammation in obesity may engage complement more prominently. Such heterogeneity underscores the need for etiology-stratified analyses in future studies (**Figure 1**).

## 5 From inflammation to sperm functional impairment: oxidative stress, microenvironment imbalance, and declining DNA integrity

### 5.1 Oxidative stress as the “mechanistic bridge”

Sperm membranes are rich in polyunsaturated fatty acids, mitochondria are metabolically active, and DNA repair capacity is limited (particularly for nuclear DNA, as mitochondrial DNA repair is even more restricted), which is thought to render sperm highly susceptible to ROS (37, 38). When myeloid amplification persists, ROS sources include both immune cell oxidative bursts and mitochondrial dysfunction in damaged tissues (39). *In vitro* studies indicate that ROS-induced lipid peroxidation reduces membrane fluidity and impairs the function of fertilization-related membrane proteins, leading to decreased motility and membrane integrity. Regarding DNA, oxidative damage increases strand breaks and base modifications, manifesting as an elevated DNA fragmentation index.

### 5.2 Dual involvement of the spermatogenic microenvironment and epididymal maturation process

Inflammation and oxidative stress cause harm at two levels. On one hand, instability of the testicular microenvironment increases germ cell apoptosis, reduces Sertoli cell supportive capacity, and lowers spermatogenic efficiency (40, 41). On the other hand, abnormal epididymal and seminal plasma environments expose sperm to higher ROS and protease loads during maturation and storage, further weakening motility and exacerbating DNA damage (42, 43).

### 5.3 SDF: inferring intervenable pathways from a “functional endpoint”

SDF can be viewed as one functional endpoint of cumulative damage inflicted on sperm by the immune-inflammatory loop, making it suitable as an “anchor point” for clinical evidence (4, 44). For example, varicocelectomy is associated with decreased DFI in several systematic reviews/meta-analyses, often accompanied by improvements in concentration, progressive motility, and morphology. This suggests its potential role in weakening the DAMP-driven inflammatory loop by reducing hypoxia/thermal stress and oxidative load.

In contrast, conclusions regarding nutritional antioxidant supplementation are inconsistent across studies. Some systematic reviews suggest certain antioxidant combinations may lead to a modest decrease in DFI (45). However, large-scale randomized, double-blind, placebo-controlled trials have shown that composite antioxidant supplementation did not improve pregnancy outcomes or key semen parameters (46, 47). Indicating that “mechanistic plausibility” does not necessarily translate into “outcome benefit”. Therefore, intervention evidence needs to be interpreted according to endpoint hierarchy.

A critical caveat in the SDF literature is the inconsistency between mechanistic plausibility and clinical outcomes. For example, while several systematic reviews suggest that varicocelectomy reduces SDF, the improvement in live birth rates remains uncertain due to the lack of large-scale, sham-controlled trials (48). Similarly, antioxidant supplementation consistently reduces oxidative stress markers in seminal plasma, but large randomized trials-such as the MOXI trial-have failed to show improvements in pregnancy or live birth (49). This disconnect highlights the need for outcome-oriented validation even when mechanistic evidence is strong.

## 6 Adaptive immunity and anti-sperm immunity: “cautious certainty” regarding clinical significance

### 6.1 Anti-sperm antibodies: a mechanistic clue, not a standalone diagnosis

Mechanistically, anti-sperm antibodies may affect sperm motility, mucus penetration, and fertilization processes. They may also impact sperm membrane structure via immune complexes or complement-mediated effects (35, 50). Clinically, ASA is better positioned as a clue along the “axis of barrier disruption and tolerance failure.” When ASA is consistent with a background of persistent inflammation, barrier damage, or dysfunctional manifestations, it is more likely to be clinically meaningful (51). When isolated and lacking association with function or outcome, over-attribution risks evidence strength challenges.

### 6.2 T Cells and immune polarity: a narrative centered on homeostasis disruption

The key significance of adaptive immunity in male infertility leans more towards “polarity regulation.” When the local environment shifts from a tolerant homeostasis toward sustained inflammation, altered immune polarity may participate in maintaining inflammation and damage (52). To maintain the convergence of a translatable narrative, this article incorporates it into the mechanistic composition of “decreased tolerance threshold and sustained inflammation” without delving into subset details difficult to close in a loop.

### 6.3 Focus for clinical translation: stratification, integration, and safety boundaries

In the absence of high-quality, outcome-oriented trial evidence, directly translating adaptive immune abnormalities into “immunotherapy”requires extreme caution (50). A more operational path is to incorporate ASA and related clues into the immune endophenotype framework to indicate the “barrier/tolerance impairment” mechanistic axis (53). Clinical management should prioritize reducing sources of ongoing damage, standardizing exclusion of infections, and integrating with ART strategies when necessary. If immune modulation is considered, principles should include short-term, monitorable, safety-first regimens, with follow-up for infection risk and hormonal axis impact (54).

## 7 Seminal immune biomarkers: clinical validation, standardized procedures, and modular interpretation

### 7.1 Why semen is an ideal window: accessibility and longitudinal monitoring advantages

Semen provides an accessible window into the immune status of the male reproductive tract. It contains immune cells, cytokines/chemokines, complement-related proteins, degranulation and NETs-related components, exosomes, and metabolism-and damage-markers related to oxidative stress (55, 56). Its advantages are non-invasiveness, repeatable sampling suitable for longitudinal monitoring, and, compared to tissue biopsy, greater alignment with the practical feasibility of clinical pathways, making it a better carrier for “process readouts” (57).

### 7.2 Standardized detection and repeated measurement: recommended SOP key points

The key challenge for seminal immune testing is not a lack of indicators but insufficient standardization of pre-analytical procedures, which may lead to non-comparable results. To enhance operability, we recommend defining and fixing several standard operating procedure(SOP) parameters (58–60).

We recommend fixing the abstinence period within the 2–7 day range and keeping it as consistent as possible during follow-up. Complete collection of the ejaculate should be recorded, including sampling time, abstinence days, and recent fever, medication, or infection symptoms. Samples should be delivered promptly, avoiding extreme temperature fluctuations, and basic processing should ideally be completed within approximately one hour after ejaculation. Liquefaction assessment should be performed within a uniform time window, for cytokine and protein detection, standardized centrifugation conditions should be used to separate seminal plasma, and repeated freeze-thaw cycles should be avoided. Whenever possible, the same detection platform and calibration process should be used for longitudinal follow-up of the same patient. Finally, contamination from lubricant, urine, or blood should be avoided, and rules should be established for handling factors such as hemolysis or high viscosity (61).

Building on this foundation, repeated measurements are recommended rather than single-point judgments: a single abnormality serves as an alert signal, whereas continuous trends guide decision-making. This approach aligns better with the clinical positioning of a “process readout” and improves reproducibility.

### 7.3 Clinical validation pathway: from correlation to predictability and intervenability

We suggest a stepwise validation pathway that moves from correlation to predictability and intervenability. The first step is to establish stable associations with functional endpoints-for example, directional links between inflammatory cell load, pro-inflammatory profiles, oxidative damage readouts, and DFI/SDF (62, 63). Once such associations are confirmed, the next step is to validate longitudinal reversibility by observing whether the immune module improves prior to functional endpoints following intervention (64). Finally, outcome-oriented prediction should be tested using endpoints such as natural conception, IVF/ICSI fertilization rates, and live birth to verify the chain of “immune module change-functional improvement-outcome improvement.” The negative outcome-level results for antioxidant supplementation highlight that even with mechanistic plausibility, reliance on outcome validation is essential to avoid over-interpretation.

Basic Panel (clinically accessible, recommended for routine configuration): This panel includes an infection/inflammation readout (seminal leukocyte count/classification or a representative pro-inflammatory marker), an oxidative damage readout (ROS or DNA oxidation damage), and a sperm functional endpoint (DFI/SDF).

Extended Panel (for research and specialized centers, suggesting unified platforms and cohorts): This panel includes complement fragments (e.g., C3a/C5a), myeloid amplification/NETs-related components, and exosome/small RNA (e.g., miRNA) multi-omics readouts. These are intended for constructing an “immune module score” and validating its predictive value for outcomes.

One unresolved issue in the field is whether seminal immune biomarkers are merely associative or truly causal in the pathophysiology of male infertility. Cross-sectional studies often report correlations between inflammatory markers and poor sperm quality, but longitudinal studies that track biomarker changes before and after intervention are still limited (65). Furthermore, no universally accepted threshold values have been established for most biomarkers, making cross-study comparisons difficult. Therefore, we propose a stepwise validation pathway that prioritizes longitudinal reversibility and outcome prediction over cross-sectional correlation (66) (**Box 2**).

Box 2 **Proposed detection panels (for endophenotype stratification and longitudinal monitoring).**

Basic Panel (clinically accessible, recommended for routine configuration): This panel includes an infection/inflammation readout (seminal leukocyte count/classification or a representative pro-inflammatory marker), an oxidative damage readout (ROS or DNA oxidation damage), and a sperm functional endpoint (DFI/SDF).

Extended Panel (for research and specialized centers, suggesting unified platforms and cohorts): This panel includes complement fragments (e.g., C3a/C5a), myeloid amplification/NETs-related components, and exosome/small RNA (e.g., miRNA) multi-omics readouts. These are intended for constructing an “immune module score” and validating its predictive value for outcomes.

## 8 Immune endophenotypes and mechanism-matched intervention: a stratified framework based on clinical evidence hierarchy

### 8.1 Clinical decision tree driven by immune endophenotypes in male infertility

Target population. Patients with a persistent “mechanistic gap” after conventional evaluation are prioritized for immune module assessment. Examples include those with only mild or moderate (or near-normal) semen parameters but impaired sperm functional endpoints or poor outcomes, such as elevated DNA fragmentation, decreased fertilization rates, poor embryo quality, or recurrent pregnancy loss.

Baseline evaluation. Before immune stratification, clinicians should complete a standard history and exposure assessment, physical examination, infection screening, imaging and hormonal evaluation, and necessary genetic tests to rule out directly localizable structural or endocrine causes or clear infections.

Standardized sampling and repeated measurement. A uniform SOP for sampling, transport, pre-processing, and platform consistency control should be followed. Repeated measurements are prioritized over single-point judgments: single anomalies serve as alert signals, whereas continuous trends guide decision-making.

Immune endophenotype determination. Integrate evidence from etiology, inflammation/myeloid amplification modules, oxidative damage readouts, and clues such as ASA to classify patients into one of four endophenotypes: Infection-Dominant, Sterile DAMP-Dominant, Barrier/Tolerance-Impaired, or Systemic Inflammation-Coupled. Mixed types are classified by the dominant axis, with allowance for re-classification during follow-up.

Mechanism-matched intervention. Follow the sequence: first remove upstream drivers, then choose paths with stronger evidence. For the Infection-Dominant type, prioritize targeted treatment with follow-up verification. For the Sterile DAMP-Dominant type, prioritize management of heat exposure, toxicants, and metabolic factors, and evaluate varicocelectomy if indicated. For the Barrier/Tolerance-Impaired type, emphasize safety boundaries and integration with ART. For the Systemic Inflammation-Coupled type, focus on lifestyle and metabolic interventions as the core.

Hierarchical efficacy evaluation and feedback. Verify across three levels: mechanistic indicators, functional indicators, and outcome indicators. First, check whether immune/oxidative modules show short-term trend improvement. Then, observe whether DNA fragmentation and function improve. Finally, validate with natural conception or ART outcomes. Failure to meet expectations triggers feedback: review the SOP, re-evaluate classification and upstream drivers, adjust the intervention, and proceed to the next round of re-testing.

### 8.2 Four endophenotypes defined by entry signal, amplification mode, and primary damage

To organize the heterogeneity of immune-mediated male infertility, we propose four immune endophenotypes. These are defined by three core dimensions: the primary entry signal (PAMP versus DAMP), the amplification mode (myeloid-driven versus inflammasome-driven), and the predominant type of damage (oxidative stress versus barrier-related impairment). Instead of relying on a single biomarker, the endophenotype classification integrates etiological clues, inflammatory readouts, oxidative damage markers, and when available, ASA status.

The first endophenotype is the infection-dominant inflammatory type. These patients show clear evidence of pathogen-related inflammation, with PAMPs as the primary trigger (67, 68). Inflammation intensity is typically high, and myeloid amplification is prominent. Clinically, such patients often respond to targeted antimicrobial therapy. In other words, the driving force is an active infectious process rather than sterile tissue damage.

The second endophenotype is the sterile DAMP-dominant inflammatory type. Here, there is no evidence of active infection. Instead, the drivers are hypoxia, thermal stress (for example, from varicocele), toxicant exposure, or local chronic injury (69). Inflammation is moderate but persistent, and oxidative damage is a prominent feature. Varicocele represents a typical example of this category. Of note, some patients with metabolic syndrome also fall into this group when sterile inflammation dominates.

The third endophenotype is the barrier/tolerance-impaired type. These patients exhibit decreased blood-testis barrier function and lowered immune tolerance thresholds. ASA may be present as a clue, but it is not a standalone diagnosis. Functional impairment tends to be persistent and less responsive to anti-inflammatory treatments alone. In other words, the core problem is not excessive inflammation but rather a failure of immune exclusion and self-tolerance.

The fourth endophenotype is the systemic inflammation-coupled type. These patients have underlying metabolic abnormalities or chronic low-grade systemic inflammation, such as obesity or insulin resistance (67). The reproductive tract inflammation resonates with systemic inflammation, requiring lifestyle and metabolic interventions as the core management strategy. For example, weight loss and improved insulin sensitivity often lead to parallel improvements in seminal immune markers.

In clinical practice, mixed phenotypes are common. When a patient meets criteria for more than one type, the dominant axis should be determined based on inflammation intensity, presence of infection, and predominance of ROS damage. Re−classification during follow−up is allowed and encouraged, as the endophenotype may shift after intervention. The proposed endophenotype classification is hypothesis-generating and currently lacks prospective validation. It should be used as a conceptual framework rather than a clinical algorithm. The main features of these four endophenotypes are summarized in **Table 1**, including their proposed biomarkers and evidence levels.

**Table 1:** Proposed immune endophenotypes in male infertility: mechanisms, biomarkers, and intervention strategies.

Endophenotype	Primary trigger	Key amplification mechanism	Representative biomarkers	Proposed intervention	Evidence level
Infection-Dominant	PAMPs (pathogens)	Myeloid amplification, TLR signaling	Seminal leukocytosis, IL-6, TNF-α	Targeted antimicrobial therapy	Clinical (observational)
Sterile DAMP-Dominant	DAMPs (hypoxia, heat, toxins, varicocele)	ROS, NLRP3 inflammasome	SDF, 8-OHdG, ROS, malondialdehyde	Remove upstream drivers (e.g., varicocelectomy), metabolic management, lifestyle modification	Clinical (RCT/meta-analysis for varicocelectomy)
Barrier/Tolerance-Impaired	BTB disruption, failure of immune tolerance	Complement cascade, autoantibody production	ASA, complement fragments (C3a, C5a)	ART integration, safety-first immunomodulation (short-term, monitorable)	Mechanistic only (animal/*in vitro*)
Systemic Inflammation-Coupled	Metabolic abnormalities (obesity, insulin resistance, dyslipidemia)	Cytokine network, systemic low-grade inflammation	IL-6, CRP, leptin, adiponectin	Lifestyle intervention, weight loss, metabolic agents (e.g., GLP-1 agonists, metformin)	Clinical (limited, mostly observational)

Evidence Level definitions:(1)Clinical (RCT/meta-analysis): High-quality human interventional studies available. (2) Clinical (observational): Human cohort or case-control studies available, but no RCT. (3) Mechanistic only: Supported by animal models and/or *in vitro* studies; human validation lacking.

This classification is hypothesis-generating and requires prospective validation in biomarker-stratified cohorts. Mixed phenotypes are common; classification should be based on the dominant mechanistic axis.

### 8.3 Intervention strategies: removing upstream drivers first, then choosing evidence-supported paths

To minimize over-extrapolation from mechanism to treatment, we present interventions for each endophenotype stratified by three endpoint levels: mechanistic endpoints (improvement in inflammation or oxidative modules), functional endpoints (improvement in SDF or motility), and outcome endpoints (natural conception or live birth). When evidence only reaches mechanistic or functional levels, we clearly state the translational boundary.

For the infection-dominant type, standardized etiological assessment and targeted antimicrobial therapy are the first priority. Follow-up testing should confirm resolution of the inflammatory module. At the mechanistic level, the goal is regression of leukocyte counts or pro-inflammatory cytokines. Subsequently, improvement in SDF or motility can be expected at the functional level. Finally, natural or ART outcomes should be validated. A key evidence gap, however, is that most studies report short-term microbiological cure rather than live birth rates.

For the sterile DAMP-dominant type, the primary strategy is to remove or mitigate upstream drivers (70, 71). This includes controlling heat exposure, smoking cessation, metabolic management, and assessing surgical indications for concurrent varicocele. The logic is to weaken the DAMP/ROS drive and oxidative load first, expecting mechanistic endpoint regression followed by functional improvement. Notably, a systematic review and meta-analysis has shown that varicocelectomy is associated with a significant reduction in SDF, representing a relatively clear example of a mechanism-matched intervention at the functional endpoint level (72). However, the same meta-analysis noted that sham-controlled trials are lacking, and the effect on live birth remains uncertain-a typical instance of the gap between mechanistic plausibility and hard outcomes.

For the barrier/tolerance-impaired type, management prioritizes reducing ongoing damage sources, standardizing infection exclusion, and integrating with ART when needed (20, 73). ASA is positioned as a mechanistic clue along the “barrier disruption and tolerance failure” axis, not as a standalone diagnosis. If immune modulation is considered, principles should include short-term, monitorable, safety-first regimens, with follow-up for infection risk and hormonal axis impact. In contrast to the previous types, anti-inflammatory treatments alone are rarely sufficient here.

For the systemic inflammation-coupled type, lifestyle and metabolic interventions are the core. The immune module serves as a process monitoring indicator to judge treatment direction via longitudinal trends. For example, a decrease in seminal IL-6 or TNF-α after weight loss can precede improvements in sperm parameters. A major unresolved issue is whether targeting systemic inflammation directly (for example, with metformin or GLP-1 agonists) improves male reproductive outcomes independently of weight loss; current evidence is mostly observational (74).

### 8.4 Hierarchical efficacy evaluation: a loop from mechanism to function to outcome

Evaluation of mechanism-matched interventions is recommended to follow a hierarchical sequence. Initially, mechanistic endpoints-such as inflammatory or oxidative modules-should show directional improvement in the short term. Subsequently, functional endpoints-exemplified by SDF, mitochondrial function, or motility-should respond. Finally, outcome endpoints-natural conception or ART outcomes-should validate clinical relevance. This sequence respects the temporal logic of the pathological process and avoids misinterpreting short-term semen parameter fluctuations as efficacy.

The importance of this hierarchical approach is underscored by the frequent dissociation between mechanistic and outcome endpoints observed in clinical studies. It is not uncommon for an intervention to improve surrogate markers-such as oxidative stress or even SDF-without translating into tangible improvements in live birth rates. This disconnect reinforces a core principle: mechanistic plausibility, while necessary, is not sufficient to guarantee clinical benefit. Therefore, efficacy assessment must be anchored in outcome-oriented validation, particularly when considering interventions with uncertain or mixed evidence profiles (75).

**Figure 2:**
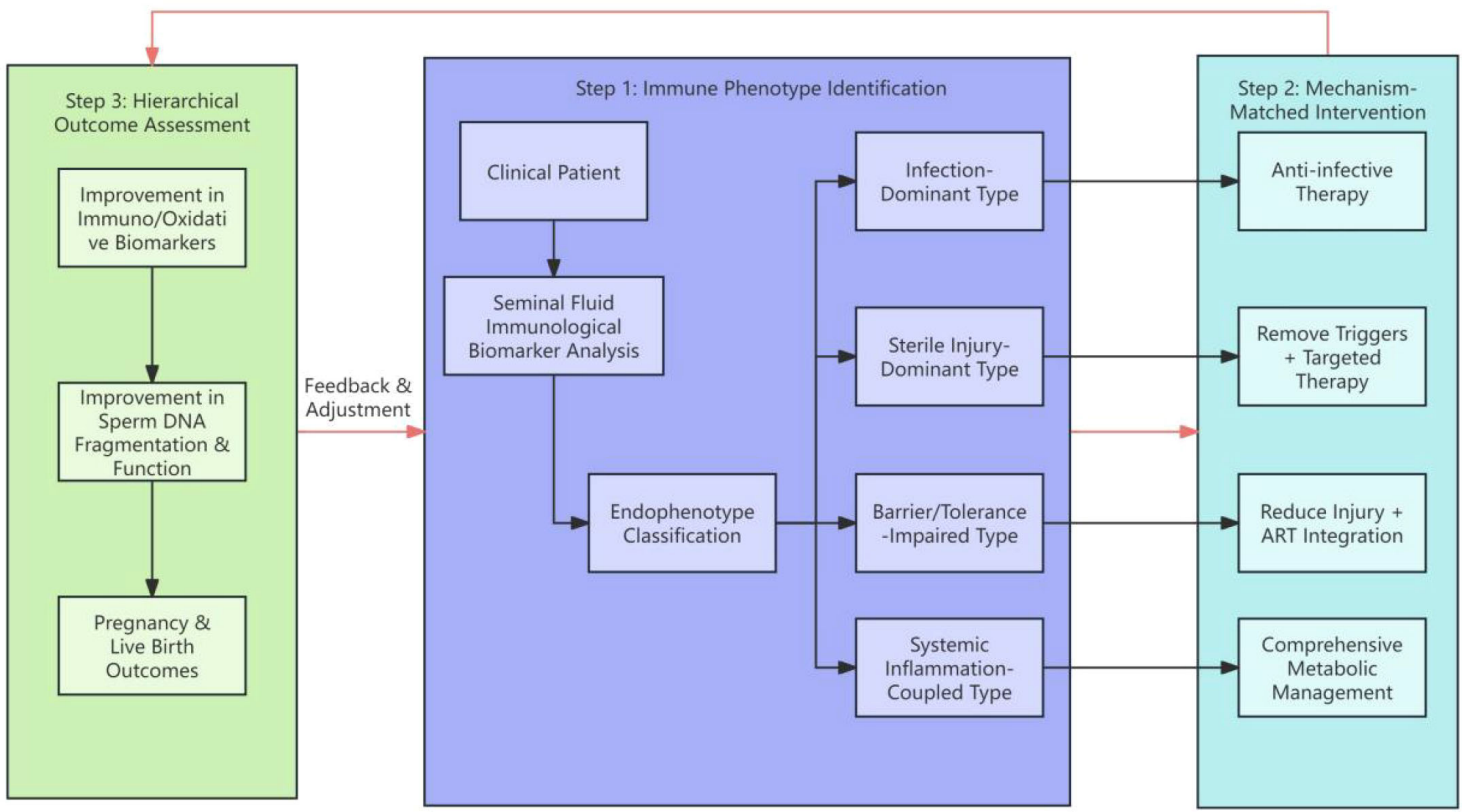
Precision management framework for male infertility based on immune endophenotypes. This clinical management framework follows a three-step logic. First (Immune Phenotyping): Analyze seminal immune biomarkers in infertile men to classify them into one of four immune endophenotypes. Second (Mechanism-Matched Intervention): Implement tailored therapeutic strategies for each endophenotype. Third (Hierarchical Efficacy Evaluation): Assess efficacy through three-level indicators (improvement in immune/oxidative markers, improvement in SDF and function, pregnancy/live birth outcomes). Failure to meet expected targets triggers a “Feedback and Adjustment” loop, prompting re-evaluation of classification and intervention strategy, forming a dynamic precision management cycle. ASA, anti-sperm antibody; ART, assisted reproductive technology.

When expected targets are not met at any level of the hierarchy, a feedback loop is triggered: review the standardized operating procedures, re-evaluate the endophenotype classification and upstream drivers, adjust the intervention, and proceed to the next round of retesting. This dynamic precision management cycle is illustrated in **Figure 2**.

## 9 Conclusion

The etiologies of male infertility are diverse, yet a convergent immune-inflammatory pathway has been proposed. Against the homeostatic background of testicular immune privilege, danger signals may trigger innate immune recognition, potentially forming an inflammatory loop sustained by myeloid amplification, complement, and inflammasomes. This loop could mutually reinforce oxidative stress, ultimately disrupting the spermatogenic microenvironment and sperm maturation process, leading to declining DNA integrity. Seminal immune biomarkers offer non-invasive and repeatable advantages, but their clinical value remains to be established through standardized SOPs, modular interpretation, and longitudinal outcome-oriented validation. Most of the evidence discussed is derived from animal models and *in vitro* studies; high-level human clinical evidence is still lacking. Future research should focus on multicenter prospective cohorts and RCTs stratified by immune endophenotypes, incorporating hard outcomes such as live birth.

### 9.1 Limitations and future directions

Future research needs to address three key challenges: establishing multicenter unified platforms and reference intervals; conducting prospective cohort and randomized controlled studies stratified by immune endophenotype; and synchronously incorporating hard endpoints like live birth with mechanistic indicators to advance male infertility management from “phenotype-based treatment” toward genuine precision medicine translation.
